# Linkage of the UK Clinical Practice Research Datalink with the national cancer registry

**DOI:** 10.1007/s10654-018-0441-5

**Published:** 2018-10-05

**Authors:** Ellena Badrick, Isabella Renehan, Andrew G. Renehan

**Affiliations:** 1 Division of Cancer Sciences, Faculty of Biology, Medicine and Health, School of Medical Sciences, Manchester Academic Health Science Centre, University of Manchester, Manchester, UK; 2 Manchester Cancer Research Centre, NIHR Manchester Biomedical Research Centre, Manchester, UK; 3 Department of Physiology, Anatomy & Genetics, University of Oxford, Oxford, UK

For some years, researchers working with primary care databases, such as the UK Clinical Practice Research Datalink (CPRD), have advocated linkage with other datasets speculating that this will enhance classification of exposures and outcomes. The article from McDonald et al. [1], focusing on CPRD, provides an updated review of studies offering empirical evidence to support the above advocacy. This is clearly illustrated with the example of classifying incident cancer. McDonald et al. [1] correctly cited the paper from Boggan et al. [2] that the level of concordance for “the recording of cancer cases between CPRD and cancer registries”—namely the National Cancer Registration and Analysis Service (NCRAS)—is 83.3% for all cancer types. However, in that study, the concordance varied by cancer type, being as low as 54% for non-melanotic skin cancers and 60% for kidney cancer. Taking this background further, Ranopa et al. [3] sought to explain why there might be differences in concordance, and through a systematic review (1998–2013), identified 84 studies with incident breast, colorectal, or prostate cancer as the diseases of interest. While the study captured data from several UK primary care databases in addition to CPRD, they demonstrated that where incident cancer was classified from GP entries (through READ codes), there was a lack of consistency in algorithms defining cancer diagnoses. For example, cancer code lists included in-situ carcinoma, and often grouped non-epithelial and epithelial malignancies from the same anatomic site as site-specific ‘cancer’. Furthermore, 27 studies used chemotherapy codes in GP records to supplement cancer classification, potentially biasing against identification of cancers where surgery or radiotherapy are the primary treatment modalities.

Dregan et al. [4] used an earlier version of CPRD (then known as GPRD), from 2002 to 2006, and determined Positive Predictive Values (PPVs) between cancer diagnoses from GPRD versus those from the national cancer registry. For the major groups of colorectal, lung, gastro-oesophageal and urological cancers, the PPVs ranged from 92 to 98%. These percentages appear reassuringly favourable but this optimism may reflect the experience of these investigators in cancer registration coding and taxonomy, and might not be generalizable to all researchers.

Given the above uncertainties and potentials for misclassification bias when studies rely soley on unlinked CPRD data, we interrogated the CPRD website publication lists and searched for the key word ‘cancer’ in titles, from 2014 to 2017 (Table 1). The primary aim was to determine the proportion of studies where CPRD was linked with cancer registries. We identified 127 papers. Of these, the outcome of interest was mainly cancer incidence; sixteen studies focused mainly on mortality. Despite the known rationale for linkage, for each of the 4 years, the proportions of reported studies that linked with the NCRAS varied from only 20–36%.

**Table 1:** Summary data for studies listed by CPRD with ‘cancer’ in their title (2014 through 2017). Proportions of studies linked with cancer registries. *Source*: https://www.cprd.com/Bibliography/Researchpapers.asp

Years of publication	No. of studies	No. of studies with linked national cancer registry (%)	No. of studies with cancer mortality as primary outcome	No. of studies with cancer diagnosis as primary outcome	No. of studies with cancer diagnosis as primary outcome linked with cancer registry
2014	36	11 (31)	5	1	0
2015	29	10 (36)	5	3	2
2016	31	6 (20)	3	1	1
2017	31	9 (31)	3	5	1
Totals	127	36 (28)	16	10	4

So why might investigative teams using primary care databases not link with national cancer registries? There are several key reasons. First, there is a considerable added administrative and logistical effort to obtain linkage between CPRD and NCRAS—typically adding 3–6 months to a project timeline. Second, the cost is approximately £10,000 (€11,000) for such a linkage. Third, historically the number of practices that link to several databases is approximately 60% of the total CPRD and period coverage for other datasets might be less than the total period covered by CPRD, ultimately reducing sample sizes. Fourth, where cancer mortality is the only cancer measure of interest, linkage with national mortality statistics (through the Office of Nations Statistics) is appropriate, without need to additionally link with NCRAS. Finally, there may be a belief that a concordance of approximately 85% is acceptable. Where the research question is one of (drug-)exposure-cancer associations, investigators might be reassured that this misclassification bias is ‘reasonable’ as associations are generally attenuated (and conservative) in this setting. However, where the research question is diagnostic with the derivation of performance characteristics (sensitivities, specificities, PPVs), we feel that this level of concordance is clinically unacceptable. Of concern, for example, in the 2015 UK National Institute for Health and Care Excellence (NICE) NG12 referral guidance for patients with symptoms suspicious for cancer [5], many studies underpinning this evidence are from analyses embedded in primary care databases but without linked cancer registry data. By illustration, none of the nine lung cancer studies (Table 4 in Ref. [5]) and none of the 31 evaluated studies in colorectal cancer (Table 21 in Ref. [5]) were linked.

It is pivotal that the epidemiology underpinning health policy decisions is minimally biased. Reflecting on the above narrative suggests that there is a serious concern of misclassification bias for cancer diagnosis in the evidence foundation of the 2015 UK NICE referral guidance. Arguably, there is a need to re-visit this evidence.
